# Risk of gastrointestinal perforation in patients taking oral fluoroquinolone therapy: An analysis of nationally representative cohort

**DOI:** 10.1371/journal.pone.0183813

**Published:** 2017-09-05

**Authors:** Shou-Chien Hsu, Shy-Shin Chang, Meng-tse Gabriel Lee, Si-Huei Lee, Yi-Wen Tsai, Shen-Che Lin, Szu-Ta Chen, Yi-Chieh Weng, Lorenzo Porta, Jiunn-Yih Wu, Chien-Chang Lee

**Affiliations:** 1 Department of Emergency Medicine, Chang Gung Memorial Hospital, Keelung, Taiwan and Chang Gung University College of Medicine, Taoyuan, Taiwan; 2 Department of Family Medicine, Taipei Medical University Hospital and School of Medicine, Taipei Medical University, Taipei, Taiwan; 3 Department of Emergency Medicine, National Taiwan University Hospital, Taipei, Taiwan; 4 Department of Rehabilitation and Physical Medicine, Taipei Veteran General Hospital, Taipei, Taiwan; 5 Department of Medicine, College of Medicine, National Yang Ming University, Taipei, Taiwan; 6 Department of Family Medicine, Chang Gung Memorial Hospital, Keelung, Taiwan and Chang Gung University College of Medicine, Taoyuan, Taiwan; 7 Department of Pediatrics, National Taiwan University Hospital Yun-Lin Branch, Douliou, Taiwan; 8 Department of Pediatrics, National Taiwan University and College of Medicine, Taipei, Taiwan; 9 Graduate Institute of Toxicology, College of Medicine, National Taiwan University, Taipei, Taiwan; 10 Dipartimento di scienze Biomediche e Cliniche, Ospedale "L. Sacco", Università degli Studi di Milano, Milan, Italy; Kaohsiung Medical University Hospital, TAIWAN

## Abstract

**Background:**

Fluoroquinolone is a commonly prescribed antimicrobial agent, and up to 20% of its users registers adverse gastroenterological symptoms. We aimed to evaluate the association between use of fluoroquinolone and gastrointestinal tract perforation.

**Methods:**

We conducted a nested case-control study on a national health insurance claims database between 1998 and 2011. The use of fluoroquinolones was classified into current (< 60 days), past (61–365 days prior to the index date) and any prior year use of fluoroquinolones. We used the conditional logistic regression model to estimate rate ratios (RRs), adjusting or matching by a disease risk score (DRS).

**Results:**

We identified a cohort of 17,510 individuals diagnosed with gastrointestinal perforation and matched them to 1,751,000 controls. Current use of fluoroquinolone was associated with the greatest increase in risk of gastrointestinal perforations after DRS score adjustment (RR, 1.90; 95% CI, 1.62–2.22). The risk of gastrointestinal perforation was attenuated for past (RR, 1.33; 95% CI, 1.20–1.47) and any prior year use (RR, 1.46; 95% CI, 1.34–1.59). To gain insights into whether the observed association can be explained by unmeasured confounder, we compared the risk of gastrointestinal perforation between fluoroquinolone and macrolide. Use of macrolide, an active comparator, was not associated with a significant increased risk of gastrointestinal perforation (RR, 1.11, 95%CI, 0.15–7.99). Sensitivity analysis focusing on perforation requiring in-hospital procedures also demonstrated an increased risk associated with current use. To mitigate selection bias, we have also excluded people who have never used fluoroquinolone before or people with infectious colitis, enteritis or gastroenteritis. In both of the analysis, a higher risk of gastrointestinal perforation was still associated with the use of fluoroquinolone.

**Conclusions:**

We found that use of fluoroquinolones was associated with a non-negligible increased risk of gastrointestinal perforation, and physicians should be aware of this possible association.

## Introduction

Gastrointestinal perforation is a lethal medical emergency that requires surgery treatment [1]. Current mortality of a perforated peptic ulcer ranges from 10 to 30% [1–4]. Gastrointestinal perforation is defined as a hole that goes all the way through the stomach, small intestine or the colon. Use of commonly prescribed pain relieving medications, such as nonsteroidal anti-inflammatory drugs, aspirin, and corticosteroids, has been correlated to an increased risk of gastrointestinal perforation [5–7]. However, to the best of our knowledge, there is no research on how other commonly prescribed drugs, such as fluoroquinolones, could cause this lethal disease.

Fluoroquinolones are one the most commonly prescribed antimicrobial agents. They are widely used in a variety of bacterial infections ranging from respiratory, abdominal, ocular, skin and skin structure, and genitourinary tract infections [8]. Part of the reasons for their widespread use are due to their broad-spectrum antibacterial coverage and excellent pharmacokinetic profiles. Although fluoroquinolones are generally well tolerated, they have been associated with a wide array of adverse events, such as tendon rupture, central nervous system effects, QT prolongation, retinal detachment, aortic dissection or aneurysm, cornea perforation and discomfort with the gastrointestinal tract. [9–16]. However, the U.S. food and drug administration only list fluoroquinolones may have adverse effects on tendons, muscles, joints, nerves, and central nervous system.[17] For other adverse effects, the U.S. food and drug administration are still assessing the evidences for public warning.

The mechanism on how fluoroquinolones can cause tendon rupture, retinal detachment, aortic dissection or aortic aneurysm has been attributed to its deleterious ability to affect collagen and connective tissues. Basic research suggests that fluoroquinolones can reduce the expression and size of type I collagen fibrils[18, 19]. Interestingly, genetic disorders with collagen deficiency, such as vascular Ehlers-Danlos syndrome, are associated with an increased risk of tendon rupture, gastrointestinal perforation and aortic dissection or aortic aneurysm [20, 21].

Since fluoroquinolones might induce collagen degradation, the gastrointestinal tract, which relies on collagen for structural integrity, might be affected. In fact, collagen pervades almost all the layers of stomach and intestinal wall, from the extracellular matrix to the serosa. We hypothesized that the use of fluoroquinolones may cause or aggravate gastrointestinal perforation with a similar mechanism to the aforementioned. To investigate this possible association, we conducted a large, nationwide, longitudinal study.

## Methods

### Setting and data collection

Under the approval of the institutional review board of the National Taiwan University Hospital, we performed a nested case-control study using the National Health Insurance Research Database (NHIRD) of Taiwan. The NHIRD database contains de-identified secondary data of approximately one million participants, and met the requirements of the “Personal Information Protection Act” of Taiwan. Thus, data were analyzed anonymously and the need for informed consent was waived. The one million participants were randomly selected from the 24 million beneficiaries of the National Health Insurance of Taiwan. Taiwan’s National Health Insurance is a government ran single-payer compulsory system, which is estimated to cover 99.6% of the entire Taiwanese population. Complete outpatient and inpatient electronic claim records, individual diagnosis, surgical procedures and prescribed medications can be found in the NHIRD database. Several studies have remarked the appropriacy of the use of the aforementioned database in pharmaco-epidemiology and drug safety researches [13, 22, 23].

### Study population

The study cohort consisted of all adults, recruited from the NHIRD and longitudinally followed from January 1998 to December 2011. We excluded both subjects younger than 18 years old on January 1^st^, 1999 and cases of appendicitis, peritonitis, and typhoid fever. Cohort members were followed from January 1^st^, 1999 until the earliest onset of one of these four possible occurrences: diagnosis of gastrointestinal perforation, termination of health insurance coverage, death or end of the study.

### Medication exposure

Use of fluoroquinolones or macrolide was assumed whenever there was any request for a reimbursement code with a prescription length ≧ 3 days. Fluoroquinolones are drugs that contain any of the following active compounds: ciprofloxacin, levofloxacin, ofloxacin, sparfloxacin, norfloxacin, lomefloxacin, moxifloxacin, gemifloxacin, enoxacin, or pefloxacin. In this study, macrolide refers to advanced macrolide that contain either one of the two following active compounds: azithromycin or clarithromycin. Index date was defined as the date of diagnosis of gastrointestinal perforation. Exposure to fluoroquinolone/macrolide was classified as ‘current’ when the most recent prescription was within 60 days from the index date, as patients with chronic bacterial prostatitis are often prescribed with long-term antibiotics (up to 6 weeks) [24]. Patients having a prescription filled between 61–365 days prior to the index date were classified as ‘past use’. ‘Any prior year use’ referred to having an antibiotic prescription that was filled for ≧ 3 days in the 1 year period before the index date. The reference category for all analyses consisted of non-fluoroquinolone/ non-macrolide use in the 1 year period before the index date.

### Outcome

Primary outcome measures were the first diagnosis of gastrointestinal perforation during the follow-up period. Gastrointestinal perforation was defined by ICD-9-CM codes of gastric perforation (531.1, 531.2, 531.5, 531.6, 532.1, 532.2, 532.5, 532.6, 533.1, 533.2, 533.5, 533.6, 534.1, 534.2, 534.5, 534.6) and small or large intestinal perforation (569.83). In sensitivity analysis, we used a more specific outcome definition by combining the aforementioned criteria with procedure codes for laparotomy or computed tomography. Current regulations in Taiwan do not allow the linkage between the NHIRD database and medical records. Thus, we tested the accuracy of our outcome definition by performing an independent validation in a tertiary medical center using one hundred electronic medical records. After review of medical records, we verified that the combined diagnostic and procedure code definition had a positive predictive rate of 89%.

### Selection of controls

For each case of gastrointestinal perforation, 100 controls were randomly selected using the incidence density sampling method and matched on index date, 5-year age group and sex. A stratum was created for each case and his/her specific controls, for a total of 1,751,000 strata. Each stratum was defined by three variables, which are: the case’s index date, 5-year age group and gender.

### Covariates

In order to be as comprehensive as possible in adjusting for factors that might confound the drug-outcome association, we reviewed literature for covariates related to gastrointestinal perforation. Table 1 shows relevant covariates for disease risk score adjustment in the following categories: demographics, index year, geographic area, annual insurance premiums, baseline comorbidities, risk factors for gastrointestinal perforation, healthcare service utilization, use of specific medications and infections within 365 days from index date. Infection is a composite covariate that is composed of 37 different common infectious diseases (S1 Table). A combined weighted comorbidity score was used to gain insights into the comorbidity differences between cases and controls, and was not used for adjustment. The combined weighted comorbidity score developed by Gagne *et al* is a summary score that combines the Charlson Index with the Elixhauser system.[25]

**Table 1:** Descriptive characteristics of patients with gastrointestinal perforation and controls.

	Cases (N = 17,510)	Controls (N = 1,751,000)	*P*-value
**Demographics**			
Gender male (%)	8646 (50.41)	864732 (50.42)	0.984
Age	53.74±18.29	53.66±18.24	0.543
**Geographic Area**			
Large central city	12641 (73.71)	1304653 (76.07)	< .0001
Mid-sized central city	3055 (17.81)	292254 (17.04)	
Suburban	1011 (5.90)	76626 (4.47)	
Countryside	442 (2.58)	41467 (2.42)	
**Annual Insurance Premiums (New Taiwan Dollars)**			
0 (Dependent)	1093 (6.37)	135683 (7.91)	< .0001
$1-$19,999	4478 (26.11)	411606 (24.00)	
$20,000-$39,999	8107 (47.27)	773186 (45.08)	
> = $40,000	3471 (20.24)	394525 (23.00)	
**Infection**			
Any infectious disease past 365 days	4080 (23.79)	98801 (5.76)	< .0001
**Comorbidity score**			
Combined comorbidity score	1.33±1.91	0.86±1.51	< .0001
**Baseline comorbidities**			
Diabetes	3506 (20.44)	281409 (16.41)	< .0001
Disease related to use of alcohol	71(0.41)	2383 (0.14)	< .0001
Disease related to use of tobacco	171 (1.00)	10931 (0.64)	< .0001
Psychiatric disorder	6216 (36.24)	425206 (24.79)	< .0001
Neurologic disorder and spinal cord injury	840 (4.90)	58586 (3.42)	< .0001
Immunocompromised states	7484 (43.64)	576677 (33.36)	< .0001
Cancer (excluding GI cancer)	1726 (10.06)	114261 (6.66)	< .0001
Benign prostatic hyperplasia	1832 (10.68)	144803 (8.44)	< .0001
Anemia	2438 (14.22)	150950 (8.80)	< .0001
Bed-ridden status	795 (4.64)	55989 (3.26)	< .0001
Obesity, diagnosed, not morbid	214(1.25)	15351 (0.90)	< .0001
Malnutrition and postgastric surgery	561 (3.27)	32364 (1.89)	< .0001
Chronic liver disease and cirrhosis	5382 (31.38)	360580 (21.03)	< .0001
Organ transplant	585 (3.41)	36407 (2.12)	< .0001
Chronic obstructive pulmonary disease	4185 (24.20)	302641 (17.65)	< .0001
Ischemic heart disease	3149 (18.36)	230416 (13.44)	< .0001
Chronic kidney disease	2876 (16.77)	202245 (11.79)	< .0001
Asthma	2599 (15.15)	178228 (10.39)	< .0001
**Risk factors for gastrointestinal perforation**			
Colorectal cancer	383 (2.23)	20813 (1.21)	< .0001
Esophageal cancer	28(0.16)	2211 (0.13)	0.2129
Stomach cancer (also called gastric cancer)	107 (0.62)	5007 (0.29)	< .0001
Inflammatory Bowel Disease (chronic)	749 (4.37)	55127 (3.21)	< .0001
Ulcerative Enterocolitis	108 (0.63)	6762 (0.39)	< .0001
Contusion With Intact Skin Surface	4877 (28.44)	379211 (22.11)	< .0001
Trauma (motor vehicle traffic accident)	117 (0.68)	9564 (0.56)	0.0295
Crushing Injury	465 (2.71)	38014 (2.22)	< .0001
Ascariasis	24 (0.14)	2127 (0.12)	0.5559
**Healthcare Service Utilization**			
Number of OPD visit	26.35±24.87	18.13±19.01	< .0001
Number of emergency department visit	0.25±1.50	0.11±0.11	< .0001
Number of hospitalization	0.31±0.98	0.17±0.87	< .0001
**Medication**			
NSAIDs	7274 (42.41)	510131 (29.75)	< .0001
Aspirin	2357 (13.37)	177158 (10.33)	< .0001
Systemic immunosuppressive agents and biologics	60 (1.69)	3496 (0.20)	< .0001
Systemic corticosteroids	2749 (16.03)	177915 (10.37)	< .0001
DMARDs	280 (1.63)	18321 (1.07)	< .0001

NSAIDs refer to Nonsteroidal anti-inflammatory drugs, and DMARDs refer to disease-modifying anti-rheumatic drugs.

S1 Fig shows the timeline for covariate collection. Chronic comorbidities and risk factors for gastrointestinal perforation were collected from year 1998 to the year before the start of the fluoroquinolone exposure period (black line). Utilization of health care facilities and use of specific medications were assessed in the one-year period preceding the year of observation for fluoroquinolone use (green line).

### Statistical analysis

Categorical variables were presented with frequency and percentage, and compared between cases and controls using Chi-squared test. Continuous variables were presented by mean ± standard deviation, and compared between cases and controls using t-test.

Under a time-matched case-control sampling scheme, the odds ratios estimate the rate ratios. Thus, we estimated the incidence rate ratios (RRs) of gastrointestinal perforation (plus 95% confidence intervals [CIs]) by three conditional logistic regressions. The first assessed the effect of fluoroquinolone use without further adjustment; the second adjusted for the Disease Risk Score (DRS) and the third matched for the DRS.

We created a study-specific DRS in the source population using the approach initially proposed by Miettinen [26, 27]. The DRS was defined as the probability of developing gastrointestinal perforation among all participants not exposed to fluoroquinolone, conditional on each individual’s baseline covariates. Operationally, DRS was calculated by a logistic regression model where gastrointestinal perforation was used as the dependent variable, while all empirical clinical predictors were treated as independent variables.

To further assess the robustness of our results, we performed an active comparator analysis, sensitivity analyses, subgroup analysis and duration response analysis. To gain insights into whether unmeasured confounders may play a role in the observed association, we conducted an active comparator analysis using a macrolide. We examined the risk of intestinal perforation for current users of macrolide as compared with non-macrolide use, and the risk of intestinal perforation for current users of fluoroquinolone as compared with non fluoroquinolone use.

In the sensitivity analysis, we investigated the effect of using different outcome definition. The codes in the sensitivity analysis can be found in S2 Table. In the subgroup analysis, we investigated whether sex or age > 70 years could be potential effect modifier. Finally, in the duration-response analysis, we categorized the cumulative duration of fluoroquinolone use into three categories (never use, light use 1–14 days, and heavy use 15–365 days) and calculated the incidence and relative risk of gastrointestinal perforation. All analyses were carried out with SAS 9.3 for Windows (SAS Institute Inc, Cary, NC), and the data were reported in accordance with STrengthening the Reporting of OBservational studies in Epidemiology (STROBE) guideline.

## Results

Table 1 summarizes the baseline characteristics of 17,510 cases of gastrointestinal perforation and 1,751,000 controls. The distribution of geographic living region, and insurance premiums were significantly different between the cases and the controls group. In general, the cases had higher number of infections, higher burden of comorbidities, higher prevalence of risk factors for gastrointestinal perforation, greater utilization of healthcare service, and greater use of medications than the control group.

### Main analyses

Table 2 shows the association between fluoroquinolone use and gastrointestinal perforation. All three types of fluoroquinolone users (current, past, or any prior-year use) had increased risk of gastrointestinal perforation, when compared with non-users. Current use of fluoroquinolone was associated with the highest unadjusted risk of gastrointestinal perforation (RR, 2.16; 95% CI, 1.85–2.53). The increase in risk of gastrointestinal perforation remained after adjusting for DRS (RR, 1.90; 95% CI, 1.62–2.22) and DRS matching (RR, 1.88; 95% CI, 1.44 2.46). The unadjusted (RR, 1.55; 95% CI, 1.41–1.71) and DRS–matched effect estimates (RR, 1.37; 95% CI, 1.17–1.60) for past use of fluoroquinolone were all attenuated compared with current use. The crude (RR, 1.69; 95% CI, 1.55–1.84) and DRS–matched effect estimates (RR, 1.48 95% CI, 1.30–1.70) for any prior-year use of fluoroquinolone fell between those of current and past use.

**Table 2:** Relationship between use of fluoroquinolones and risk of gastrointestinal perforation.

	FQ usage rates for all the perforation cases	FQ usage rates for all the controls	Effect estimate matched on age group, gender, and year (RR, 95% confidence interval)	Effect estimate adjusted by disease risk score (RR, 95% confidence interval)	Effect estimate matched by disease risk score (RR, 95% confidence interval)
Current use (1–60 day)	166/17150 (0.96%)	7809/1715000 (0.46%)	2.16 (1.85–2.53)***	1.90 (1.62–2.22)***	1.88 (1.44 2.46)***
Past use (61–365 day)	411/17150 (2.40%)	26960/1715000 (1.57%)	1.55(1.41–1.71)***	1.33 (1.20–1.47)***	1.37 (1.17 1.60)***
Any prior-year use (1–365 days)	577/17150 (3.36%)	34769/1715000 (2.03%)	1.69(1.55–1.84)***	1.46 (1.34–1.59)***	1.48 (1.30 1.70)***

*** refers to p<0.001

RR refers to rate ratio.

### Active comparator analysis

To gain insights into whether the observed association could be explained by unmeasured confounders, we compared the risk of gastrointestinal perforation between fluoroquinolone and macrolide, a similar broad-spectrum antibiotic (Table 3). Use of fluoroquinolone was associated with an increased risk of gastrointestinal perforation after DRS adjustment (RR, 1.90, 95%CI, 1.62–2.22). In contrast, use of macrolide, an active comparator, was not associated with a significant increase risk of gastrointestinal perforation after DRS adjustment (RR, 1.11, 95%CI, 0.15–7.99).

**Table 3:** Sensitivity analysis using macrolide as an active comparator.

	Effect estimate adjusted by disease risk score (RR, 95% confidence interval)
Current fluoroquinolone use (1–60 day)	1.90 (1.62–2.22)***
Current macrolide use (1–60 day)	1.11 (0.15–7.99)

*** refers to p<0.001

RR refers to rate ratio.

### Risk of perforation associated with different anatomic sites

There are architectural differences between stomach and intestines. Thus, we investigated whether fluoroquinolone could differentially affect the risk of gastric and intestinal perforation. (Table 4) We found that for current use of fluoroquinolone, gastric perforation and small or large intestinal perforation have similar unadjusted and adjusted risk of perforation. However, the effect estimates for small or large intestinal perforation have generally larger confidence interval and are not statistically significant.

**Table 4:** Relationship between current use of fluoroquinolones and risk of perforation associated with different anatomic sites.

	Effect estimate matched on age-group, gender, and year (RR, 95% confidence interval)	Effect estimate adjusted by disease risk score (RR, 95% confidence interval)	Effect estimate matched by disease risk score (RR, 95% confidence interval)
Gastric perforation	2.18 (1.86–2.55)***	1.92 (1.63–2.25)***	1.85 (1.42–2.43)***
Small or large intestinal perforation	1.84 (0.87–3.90)	1.44 (0.64–3.24)	3.00(0.57–15.96)

*** refers to p<0.001

RR refers to rate ratio.

### Sensitivity analyses

In order to verify the robustness of the primary results, and examine the various effects of outcome definitions, we repeated the primary analyses on different outcome definitions (Table 5). In all outcome definitions, a higher risk of gastrointestinal perforation was associated with use of fluoroquinolone. This was also true in the strictest definition for gastrointestinal perforation, where only those who had undergone operations for gastrointestinal operations were included. To mitigate selection bias, we have also excluded people who have never used fluoroquinolone before or excluded people with infectious colitis, enteritis or gastroenteritis. In both of analyses, a higher risk of gastrointestinal perforation was still associated with use of fluoroquinolone.

**Table 5:** Sensitivity analysis using different outcome definition for current use of fluoroquinolone.

	Effect estimate matched on age-group, gender, and year (RR, 95% confidence interval)	Effect estimate adjusted by disease risk score (RR, 95% confidence interval)
Gastric perforation only (N = 159)	2.18 (1.86–2.55)***	1.92 (1.63–2.25)***
Gastric perforation undergoing surgery only (N = 394)	1.56 (1.41–1.73)***	1.33 (1.20–1.48)***
Small or large intestinal perforation only (N = 7)	1.84 (0.87–3.90)	1.44 (0.64–3.24)
Small or large intestinal perforation undergoing surgery only (N = 17)	1.33 (0.82–2.16)	1.24 (0.76–2.02)
Gastrointestinal perforation excluding people who never use fluoroquinolone before (N = 166)	1.51 (1.19–1.91)***	1.54 (1.21–1.96)***
Gastrointestinal perforation excluding people with infectious colitis, enteritis or gastroenteritis (N = 165)	2.18 (1.86–2.54)***	1.91 (1.63–2.23)***

*** refers to p<0.001

RR refers to rate ratio.

### Subgroup analysis

To further assess the robustness of our results, we stratified the study population based on >70 years of age and gender (Table 6). We found that the increase in risk of gastrointestinal perforation in current user of fluoroquinolones was not substantially affected by age or gender.

**Table 6:** Subgroup analysis of the relationship between current use of fluoroquinolones and risk of composite gastric or intestinal perforation.

	Patient subgroups	Disease Risk Score adjusted RR (95% Confidence interval)
Fluoroquinolone user v.s. non-user	>70 years of age	1.94 (1.49–2.52)***
	< = 70 years of age	1.88 (1.55–2.29) ***
	Male	1.84 (1.45–2.32) ***
	Female	1.94 (1.57–2.40) ***

*** refers to p<0.001

RR refers to rate ratio.

### Duration-response analysis

To gain an insight into whether increasing use of fluoroquinolone might lead to an increase risk of gastrointestinal perforation, we carried out a duration response analysis. On Table 7, we used non-user of fluoroquinolone as a reference. We found that both the crude incidence rate and the DRS adjusted rate ratio increased with the duration of fluoroquinolone use.

**Table 7:** Duration-response analysis.

Cumulative duration of fluoroquinolone use	Incidence and risk of perforation by duration of fluoroquinolone use in the 60-day risk period	
	IR % (case/person-years)	Disease Risk Score adjusted RR (95% Confidence interval)
0 days (reference)	0.99% (16984/1724168)	Reference
1–14 days	1.95% (50/2,558)	2.01 (1.52–2.66) *
>14 days	2.14% (144/5424)	2.20 (1.83–2.65) ***

* refers to p<0.05, and

*** refers to p<0.001

RR refers to rate ratio.

## Discussion

In this population-based study, we found that current use of fluoroquinolone was associated with an approximately 2-fold increased risk of gastrointestinal perforation. Any prior-year use were similarly associated with an increased, although attenuated, risk for these severe adverse events. We also found that longer durations of fluoroquinolone therapy were associated with a higher incidence of gastrointestinal perforation. The risk increase of gastrointestinal perforation was more substantial in patients older than 70 years and in male patients.

The global consumption of fluoroquinolones was estimated to increase from 4.75 billion doses to 7.81 billion doses, from year 2000 to 2010 [28]. In the United States alone, fluoroquinolone prescription during physician visits has increased by approximately 3 folds between 1995 and 2002, from about 7 million to 22 million prescriptions. For Taiwan, users of fluoroquinolone have increased approximately by 1.8 folds during this study period, from 0.5 million to 0.9 million users [13]. With the rapid increase in the consumption of fluoroquinolones, more rare adverse events are being reported. Uncommon adverse events with fluoroquinolones include tendinitis or tendon rupture, retinal detachment, aortic aneurysm or dissection, seizure, QT interval prolongation, hepatotoxicity, and dysglycemia [9–13, 29–32].

Among the uncommon adverse events, the risks of tendinitis and tendon rupture have been studied the most. In a recent systematic review, use of fluoroquinolones was associated with a 3–4 folds increase in the risk of tendinitis, and a 2–7 folds increase in risk of tendon rupture [33]. Our findings were concordant with the lower limit observed for tendon rupture. As for the risk period, case control studies suggest that use of fluoroquinolone within 90 days of the indexed tendon rupture has the greatest risk. Yan der Linden et al. found that the risk of tendon rupture decreased from 4.3 fold to 1.4 fold when the risk period for fluoroquinolone use changed from 30 days to 7–18 months [34]. Corrao et al. also showed that the risk of tendon rupture decreased from 4.1 fold to 1.1 fold when the risk period for fluoroquinolone use changed from 15 days to 1–3 months [35]. Our results, demonstrating a higher risk of gastrointestinal perforation within 60 days of fluoroquinolone therapy, are in concordance with the aforementioned studies.

While the exact mechanism on how use of fluoroquinolone might result in gastrointestinal perforation is unknown, there are several plausible mechanisms on how fluoroquinolone can affect the synthesis or structural integrity of collagen, major structural protein of the connective tissues of the gastrointestinal tract [36, 37]. First, fluoroquinolones have chelating properties against several metal ions (e.g., calcium, magnesium, aluminum), and exhibit a higher affinity for those in the connective tissue than those measured in serum [38–40]. Metal ions are essential for type 1 collagen synthesis, thus fluoroquinolone may promote collagen degradation by chelating the aforementioned metal ions [9, 40, 41]. Second, fluoroquinolones have been shown to result in cartilage damage by inducing necrosis of chondrocytes (36 hours after treatment), disruption of the extracellular matrix, and formation of vesicles and fissures at the articular surface [42]. Finally, Fluoroquinolones have been found to decrease collagen synthesis by increasing the expression of matrix metalloproteinases, which have the ability to degrade collagen [43–45]. Specifically, fluoroquinolones have been found to up-regulate the expression of matrix metalloproteinases (MMP)-2 and MMP-3 in tendon cells, and MMP-1, MMP-2, MMP-8 and MMP-9 in cornea cells [18, 44, 46, 47]. MMP-1, MMP-2, MMP-3 and MMP-9 are also found in the gastrointestinal tract, and the up-regulation of these MMPs have been associated with gastrointestinal tract ulceration or perforation [48–50].

Interestingly, there are several reports associating corneal perforation with fluoroquinolones therapy [15, 16, 51]. Gangopadhyay et al. found that in 140 patients with bacterial corneal ulcers, administration of fluoroquinolone therapy is associated with 9.3% perforation cases. However, none of the corneal ulcers patients treated with combined fortified antibiotics (tobramycin 1.3% and cefazolin 5%) developed the perforation complication [51]. Using a different patient cohort, Mallari *et all* found that in 270 patients with bacterial keratitis, the incidence of corneal perforations was 18 fold higher in the keratitis patients that were treated with fluoroquinolone as compared with patients treated with fortified antibiotics (12.7% vs 0.7%). In our study, we found that the unadjusted rate of perforation is around 0.99% in patients not prescribed with any fluoroquinolone, 1.95% in patients prescribed with 1–14 days of fluoroquinolone, and 2.14% in patients prescribed with greater than 14 days of fluoroquinolone in a calendar year.

Results of this study should be interpreted in light of both strengths and limitations. Using a nationally representative database with large number of participants is a major strength of this study. In fact, the nationally representative database ensured minimal risk of selective population and related potential bias. In addition, the large number of gastrointestinal perforation cases gave us better statistical power for analysis using different exposure categories, and covariate adjustments.

Our study also has inherent limitations. First, unmeasured confounders are always present in claims database, in fact many life style factors, such as alcohol drinking and smoking, are missing. Both of these factors may increase the risk of gastrointestinal perforation. Thus, we used alcohol or smoking-related diseases as a proxy for confounding adjustment. In addition, we conducted an active comparator analysis to gain insights into whether unmeasured confounders may play a role in the observed association. We found that use of macrolide, was not associated with a significant increase risk of gastrointestinal perforation. Second, the possibility of protopathic bias, interpreted as symptoms preceding the diagnosis of gastrointestinal perforation leading to prescription of fluoroquinolones, cannot be totally ruled out. To minimize the risk of protopathic bias, we identified three possible indication of fluoroquinolone therapy (appendicitis, peritonitis and typhoid fever) that could be associated with gastrointestinal perforation and excluded them from our analysis. Third, selection bias cannot be totally excluded. To mitigate selection bias, we also excluded people who have never use fluoroquinolone before or people with infectious colitis, enteritis or gastroenteritis in our sensitivity analysis. In both of the analysis, a higher risk of gastrointestinal perforation was still associated with use of fluoroquinolone. Third, this study did not exclude prior users of fluoroquinolone, which may underestimate the risk of perforation. Past studies have found that new drug users tend to have a stronger adverse effect, due to the phenomena of depletion of susceptible effect.[52] Finally, we adopted the common time-window approach to assess drug exposure instead of using the duration-specific approach.[53] The duration-specific approach may give a more unbiased result, but is much more computational intensive, and may require super computer for a large dataset like this. Thus, future studies using different drug exposure methods or study designs are required to validate our result.

Even though our study design cannot establish a direct cause and effect relationship, it is not likely that more detailed information on a larger population will become available in the immediate future. Given the high mortality associated with gastrointestinal perforation, these findings may warn the clinicians to weigh the overall risk-benefit balance of fluoroquinolone treatment in patients at high risk for gastrointestinal perforation.

## Supporting information

S1 Table ICD-9-CM codes for infectious disease. (DOCX)

S2 Table ICD-9-CM or procedure codes in sensitivity analysis. (DOCX)

S1 Fig Timeline for covariate collection. (DOCX)
