# Supplementary material for: Risk of gastrointestinal perforation in patients taking oral fluoroquinolone therapy: An analysis of nationally representative cohort
Source: PLoS One. 2017 Sep 5;12(9):e0183813. doi: 10.1371/journal.pone.0183813 (PMC5584983; doi:10.1371/journal.pone.0183813)
Supplement: S1 Table — (DOCX) [file pone.0183813.s001.docx]

**S1 Table:** ICD-9-CM Codes for infectious disease

| **Disease** | **ICD-9-CM Codes** |
| --- | --- |
| Infectious colitis, enteritis, and gastroenteritis | 009.0 |
| Infectious diarrhea | 009.2 |
| Tuberculosis of meninges and central nervous system | 013 |
| Tuberculosis peritonitis | 014 |
| Tuberculosis of bones and joints | 015 |
| Tuberculosis of genitourinary system | 016 |
| Tuberculosis of other organs | 017 |
| Miliary tuberculosis | 018 |
| Bacterial meningitis | 320 |
| Acute and subacute bacterial endocarditis | 421.0 |
| Acute and subacute infective endocarditis in diseases classified elsewhere | 421.1 |
| Acute sinusitis | 461 |
| Acute bronchitis and bronchiolitis | 466 |
| Pneumococcal pneumonia | 481 |
| Other bacterial pneumonia | 482 |
| Pneumonia due to other specified organism | 483 |
| Pneumonia in infectious diseases classified elsewhere | 484 |
| Bronchopneumonia, organism unspecified | 485 |
| Pneumonia, organism unspecified | 486 |
| Bronchitis, not specified as acute or chronic | 490 |
| Chronic bronchitis | 491 |
| Bronchiectasis | 494 |
| Peritonitis and retroperitoneal infections | 567 |
| Acute cholecystitis | 575.0 |
| Other cholecystitis | 575.1 |
| Cholangitis | 576.1 |
| Infections of kidney | 590 |
| Urinary tract infection, site not specified | 599.0 |
| Carbuncle and furuncle | 680 |
| Cellulitis and abscess of finger and toe | 681 |
| Other cellulitis and abscess | 682 |
| Acute lymphadenitis | 683 |
| Impetigo | 684 |
| Pilonidal cyst | 685 |
| Other local infections of skin and subcutaneous tissue | 686 |
| Osteomyelitis periostitis and other infections involving bone | 730 |
| Fever and other physiologic disturbances of temperature regulation | 780.6 |
