# Supplementary material for: Risk of gastrointestinal perforation in patients taking oral fluoroquinolone therapy: An analysis of nationally representative cohort
Source: PLoS One. 2017 Sep 5;12(9):e0183813. doi: 10.1371/journal.pone.0183813 (PMC5584983; doi:10.1371/journal.pone.0183813)
Supplement: S2 Table — (DOCX) [file pone.0183813.s002.docx]

S2 Table. ICD-9-CM or procedure codes in sensitivity analysis

| **Disease** | **Codes** |
| --- | --- |
| **Gastric perforation only** | 531.1, 531.2, 531.5, 531.6, 532.1, 532.2, 532.5, 532.6, 533.1, 533.2, 533.5, 533.6, 534.1, 534.2, 534.5, 534.6 |
| **Gastric perforation undergoing surgery only** | 531.1, 531.2, 531.5, 531.6, 532.1, 532.2, 532.5, 532.6, 533.1, 533.2, 533.5, 533.6, 534.1, 534.2, 534.5, 534.6 and 569.83 and [laparotomy (54.1, 75805B, 75809B**)** or computed tomography (33070B, 33071B, 33072B)] |
| **Small or large intestinal perforation** | 569.83 |
| **Small or large intestinal perforation** | 569.83 and [laparotomy (54.1, 75805B, 75809B**)** or computed tomography (33070B, 33071B, 33072B)] |
| **Infectious colitis, enteritis or gastroenteritis** | 009.0, 009.1, 556.0 |
