# Supplementary figures and images for: Risk of gastrointestinal perforation in patients taking oral fluoroquinolone therapy: An analysis of nationally representative cohort
Source: PLoS One. 2017 Sep 5;12(9):e0183813. doi: 10.1371/journal.pone.0183813 (PMC5584983; doi:10.1371/journal.pone.0183813)

S1 Figure. Timeline for covariate collection.

**
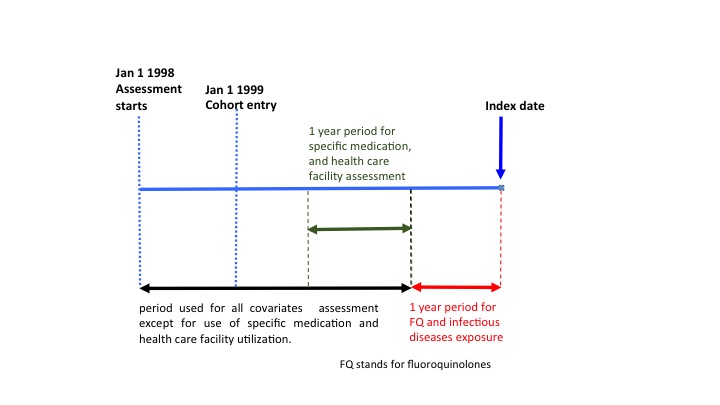
**

Supplement: S1 Fig — (DOCX) [file pone.0183813.s003.docx]
